# Endogenous sex steroid hormones, sex hormone binding globulin and risk of all-cause and cardiovascular mortality in patients with established cardiovascular diseases: a systematic review and meta-analysis of prospective studies

**DOI:** 10.3389/fendo.2026.1878347

**Published:** 2026-07-27

**Authors:** Mojgan Amiri, Hamidreza Raeisi-Dehkordi, Sara Beigrezaei, Angeline Chatelan, Oscar H. Franco, Trudy Voortman, Taulant Muka

**Affiliations:** 1 Department of Epidemiology, Erasmus MC University Medical Center, Rotterdam, Netherlands; 2 Department of Global Public Health and Bioethics, Julius Center for Health Sciences and Primary Care, University Medical Center (UMC) Utrecht, Utrecht University, Utrecht, Netherlands; 3 Geneva School of Health Sciences, HES-SO University of Applied Sciences and Arts Western Switzerland, Geneva, Switzerland; 4 Epistudia, Bern, Switzerland; 5 Research Department, Swiss TCM University, Bad Zurzach, Aargau, Switzerland

**Keywords:** CVD, death, mortality, sex steroid hormones, SHBG

## Abstract

**Background:**

Endogenous sex steroid hormones are involved in numerous regulatory mechanisms of the cardiovascular system and their imbalances are frequently observed in patients with established cardiovascular disease (CVD). However, their prognostic significance for mortality remains unclear. This study aims to systematically synthesize existing research and quantify the association between endogenous sex steroid hormones, sex hormone binding globulin (SHBG), and the risk of all-cause and cardiovascular mortality in individuals with established CVD.

**Methods:**

Six bibliographic databases were systematically searched. Pooled hazard ratios (HRs) and 95% confidence intervals (CIs) were calculated using a random-effects model, comparing the highest versus lowest levels of sex hormones/SHBG. The risk of bias was evaluated using the ROBINS-E tool (Risk Of Bias In Non-randomized Studies - of Exposures).

**Results:**

Twelve studies with a total of 5,981 patients with established CVD were included. No significant association was found between endogenous total testosterone and risk of all-cause ((HR (95%CI): 0.78 (0.56 to 1.09), n= 5 studies) or CVD (HR (95%CI): 1.30 (0.28 to 6.01), n= 3) mortality in men. Most studies (seven out of twelve studies) were classified as having a high risk of bias, particularly due to confounding.

**Conclusions:**

We found no evidence for an association between endogenous sex hormones and mortality outcomes in patients with CVD. This study underscored the lack of sufficient evidence on this topic, especially concerning women.

**Systematic Review Registration:**

https://www.crd.york.ac.uk/PROSPERO/view/CRD42022329605, identifier CRD42022329605.

## Introduction

Endogenous sex steroid hormones and sex hormone-binding globulin (SHBG) play essential roles in several physiological processes and have been implicated in the development of chronic conditions such as obesity, insulin resistance, type 2 diabetes, and cardiovascular disease (1–13). Additionally, these hormones and SHBG have been associated with mortality risk in the general population (13, 14), with sex-specific patterns observed in previous meta-analyses (14). However, evidence on the relationship between endogenous sex steroid hormones, SHBG, and mortality risk in individuals with cardiovascular disease (CVD) remains limited.

This gap is particularly important given that hormonal imbalances have been reported in patients with CVD (15, 16), and previous studies examining the association between these hormones and mortality in this population have produced inconsistent findings. Some studies in men suggest that lower testosterone levels are associated with an increased risk of mortality in patients with coronary heart disease (17) and heart failure (18). However, no significant associations have been observed in male patients with acute coronary syndromes (19) or in men with systolic chronic heart failure (20). While one study found that higher estradiol levels are associated with all-cause mortality in men referred to angiography (21), another study showed that both high and low estradiol levels are associated with risk of mortality in men with systolic chronic heart failure (22).

Furthermore, evidence on the association between endogenous sex steroid hormones and mortality risk in women with cardiovascular disease is scarce and remains inconclusive. Some studies have reported positive associations; for example, between estradiol and short-term mortality in postmenopausal women with acute stroke (23), and between higher SHBG levels and all-cause mortality in women at high cardiovascular risk (24). However, other studies found no significant associations for SHBG or dehydroepiandrosterone sulfate (DHEAs) in women with acute stroke (25). Given the limited and inconsistent evidence, this systematic review and meta-analysis aims to synthesize existing studies and quantify the association between endogenous sex steroid hormones, SHBG, and mortality risk in individuals with established CVD.

## Methods

This systematic review was designed and carried out in accordance with two recent guidelines for systematic reviews, and the findings are reported following the PRISMA recommendations (27–29). The study protocol was registered in PROSPERO (CRD42022329605) as part of a larger systematic review project; this manuscript focuses on patients with established CVD.

### Data sources and search strategy

To identify potentially relevant studies, we combined keywords related to sex hormones and mortality. We conducted searches in Embase.com, MEDLINE ALL (Ovid), Web of Science Core Collection, Cochrane Central, and PubMed (publisher), with the search updated until February 26, 2025. Additionally, the first 200 hits from Google Scholar were included. Additionally, we applied filters to exclude conference abstracts, case reports, studies involving non-adult populations, and animal studies. The search strategy was developed by an expert research librarian. The search results were imported into EndNote and de-duplicated using the method outlined by Bramer et al. (26). The specific details of the search strategy and keywords are presented in **Supplementary Table 1**. To further enhance our search, we manually reviewed the references of the included studies and relevant published reviews.

### Eligibility criteria

We included original studies that met the following criteria: (i) they had an observational prospective design; (ii) they were conducted in a patients with established cardiovascular disease; (iii) they measured at least one of the following exposures: total testosterone, bioavailable testosterone, free testosterone, dehydroepiandrosterone (DHEA), DHEAs, and estradiol, or SHBG; (iv) they reported associations with all-cause or cause-specific mortality, including CVD, cancer, and other causes; and (v) they provided adjusted effect estimates (relative risk (RR), hazard ratio (HR), or odds ratio (OR) with 95% confidence intervals (95% CI).

We excluded retrospective studies, conference abstracts, ecological studies, case reports, case series, letters to the editor, conference proceedings, narrative reviews, systematic reviews, and meta-analyses. Studies conducted on animals, children, or adolescents were also excluded. In addition, we excluded studies focusing on other chronic diseases, studies conducted in the general population, and studies that did not clearly specify that participants were diagnosed with cardiovascular disease.

### Study selection and data extraction

All titles and abstracts were screened in duplicate by two independent teams of researchers (MA, HRD, and SB) based on the eligibility criteria. Subsequently, the selected full texts were reviewed again in duplicate by MA and HRD. Data from the included studies were extracted using a pre-designed form created by MA, HRD and SB. The extracted information included the first author’s name, study design, study title, established type of CVD, publication year, location, number of participants, sex distribution of the population, age, follow-up duration, levels of sex hormones, methods of sex hormone assessment, number of deaths, causes of mortality, adjustments, and hazard/risk/odds ratios.

### Risk of bias assessment

Risk of bias assessments were conducted by HRD, MA, and SB, with any discrepancies resolved through consensus. The risk of bias for cohort studies was evaluated using the latest version of the ROBINS-E tool (Risk Of Bias In Non-randomized Studies - of Exposures) (26). This tool provides a structured approach to assessing the risk of bias in observational epidemiological studies and shares many characteristics with the risk of bias (ROB) 2 tool for randomized clinical trials and the ROBINS-I tool for non-randomized intervention studies. Indeed, it provides further development of ROBINS-I. The tool includes 7 domains and considers potential biases resulting from confounding, assessment of exposure, selection of participants, post-exposure interventions, missing data, measurement of the outcome, and selective reporting of the results (26).

### Statistical analysis

We used the random-effects model by Hartung-Knapp method for small number of studies (e.g., ≤5), which considers both between-study and within-study variations (heterogeneity), to calculate the summary HR and 95% CI of all-cause and cause-specific mortality for the highest and lowest categories of sex hormones. In the analysis, we used the most extensively adjusted HRs reported. For studies that only reported risk estimates on a continuous scale, if possible, we converted the HRs per-unit increment to the highest and lowest level of exposure using the average difference between the midpoints of the upper and lower categories of that relevant study in the analysis, as done in previous studies (27, 28). Heterogeneity between studies was assessed using the I^2^ (29). Sensitivity analysis was performed to explore if the results were robust using leave-one-out analysis, excluding each study at a time from analysis. We performed the statistical analyses of our meta-analysis in R (Version 4.0.5) using meta, dmetar, and dosresmeta packages. P <0.05 was considered significant.

## Results

### Eligible studies

Out of 13,135 references screened, twelve studies were included in the systematic review. Among included studies, eight studies examined the association between total testosterone levels and the risk of death in populations with established CVD (19, 20, 22, 30–34), with only six studies in men providing sufficient data to be included in the meta-analysis of total testosterone with the risk of all-cause and CVD mortality. One study was excluded due to insufficient data for estimate conversion (31), and another was conducted among women (30). The rest of the studies (n=6) were included only in the narrative analysis (23, 30, 31, 35–37).

**Figure 1:**
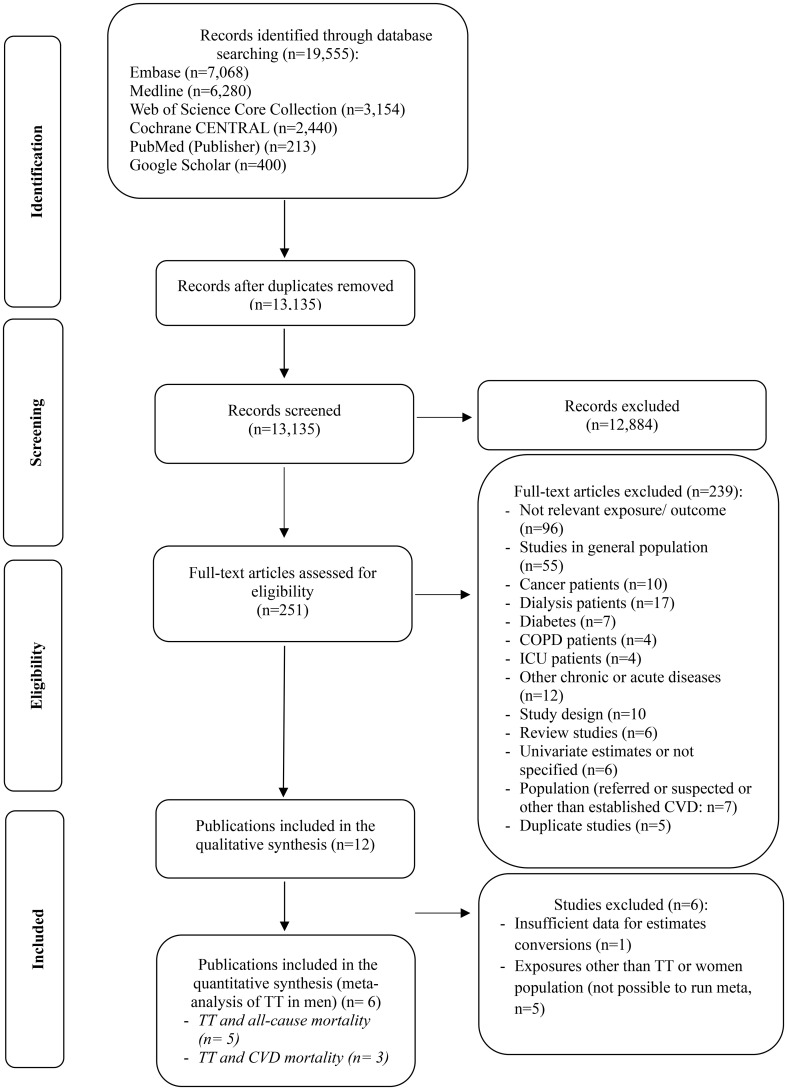
Study selection procedure.

A detailed outline of the study selection process is presented in **Figure 1**.

### Study characteristics and assessment of risk of bias

The included studies were published between 2009 and 2023 and comprised a total of 5,981 patients with established CVD, among whom 709 deaths were due to all causes and 151 were attributed specifically to CVD (**Table 1**). We found no studies reporting on death due to other causes or cancer as the outcomes.

**Table 1:** Characteristics of the included studies in the systematic review.

Study				Participants				Exposures	Outcomes			
Author, year	Country	Design	Follow-up	Sex	Health status	Age	No. of participants	Sex hormones	Measurement methods	All-cause	CVD	Adjustments
Marra et al., 2023 (30)	Italy	Prospective cohort	3 y	F	HF	66.2 ± 10.3	94	TT	Immunoassay	18	–	Age, NYHA class, LVEF, impaired eGFR, NT-proBNP, haemoglobin levels, aetiology, HF duration, and peak VO2.
Raparelli et al., 2022 (36)	Italy	Prospective cohort	23.7 m	B	Ischemic heart disease	69.8 ± 11.5	434	Ratio TT/E2	Chemiluminescent microparticle immunoassay	41	–	Sex, ACS presentation, obstructive type of CAD.
Gencer et al., 2021 (19)	Switzerland	Prospective cohort	1 y	M	ACS	Min: 18	1054	TT	ECL	42	35	Global Registry of Acute Coronary Events risk score (composite of age, heart rate, SBP, creatinine, cardiac arrest at admission, ST segment deviation, abnormal troponin enzyme and Killip classification), preexisting DM and inflammation (hs-CRP).
Boden et al., 2020 (32)^*^	USA	Prospective cohort	3 y	M	Atherosclerotic CVD	40	2118	TT	Liquid chromatography coupled with tandem mass spectrometry.	109	55	Age, BMI, history of angina, DM, and HTN.
Yoshihisa et al., 2018 (31)	Japan	Prospective cohort	3.5 y	M	HF	65.9	618	TT	ECL	180	–	Age, BMI, NYHA class III or IV, LVEF, ischemic etiology, HTN, DM, dyslipidemia, CKD, anemia, atrial fibrillation.
Pesonen et al., 2016 (34)^**^	Sweden	Prospective cohort	5.6 y	M	ACS	62.3 ± 9.4	264	TT	Radioimmunoassay	–	45	Age, current smoking, cholesterol, CRP, weight.
Pappa et al., 2012 (23)	Greece	Prospective cohort	30 d	F	Acute stroke	73.6 ± 12.9	302	E2	Spectria E2-sensitive radioimmunoassay	25	–	Arterial hypertension, National institutes of health stroke scale
Wu et al., 2011 (20)	China	Prospective cohort	3.4 y	M	Systolic CHF	68.5 ± 5.4	175	TT, FT, SHBG	TT: ECL; FT: Vermeulen equation; SHBG: ELISA	54	–	Age, NYHA class, LVEF, SBP, comorbidities, medications, NT-proBNP, e-GFR, plasma albumin, hemoglobin.
Guder et al., 2010 (33)	Germany	Prospective cohort	859 d	M	HF	64 ± 13	191	TT, FT, DHEAs, SHBG	TT, DHEAs, SHBG: Immunoassays; FT: Vermeulen equation.	53	–	Age, NYHA class, renal function, atrial fibrillation, SBP, CRP, N-terminal pro-brain natriuretic peptide (NT-proBNP), intake of diuretics, cortisol.
Militaru et al., 2010 (35)	Romania	Prospective cohort	30 d	M	Acute MI	61.7 ± 12.9	126	FT	Chemoluminiscence assay	16	–	Age, BMI, LDL and HDL cholesterol, TG, cigarette moking, DM, history of HTN, LVEF, hs-CRP and NT-proBNP.
Pascual-Figal et al., 2009 (37)	Spain	Prospective cohort	3 y	M	HF	53.1 ± 10.6	104	SHBG	Radioimmune test	–	16	Age, BMI, spironolactone, loop diuretics.
Jankowska et al., 2009 (22)	Poland	Prospective cohort	3 y	M	Systolic CHF	58 ± 12	501	E2, TT, DHEAs	E2: ECL; TT, DHEAs: Immunoassays.	171	–	Age, BMI, NYHA class, HF etiology, LVEF, plasma NT-proBNP, serum sodium, hemoglobin, estimated GFR, plasma total cholesterol, serum uric acid, therapy with loop diuretic, therapy with spironolactone, SBP, history of HTN, DM, and smoking

y, Years, d, Days; m, Months; M, male; F, Female; B, Both; TT, Total testosterone; FT, Free testosterone; DHEAs, Dehydroepiandrosterone sulfate; SHBG, Sex hormone-binding globulin; E2, Estradiol; CVD, Cardiovascular diseases; ACS, Acute coronary syndrome; BMI, Body mass index; HF, Heart failure; CHF, Chronic heart failure; MI, Myocardial infarction; DM, Diabetes mellitus; HTN, Hypertension; CRP, C-reactive protein; SBP, Systolic blood pressure; TG, triglycerides; NT-proBNP, N-terminal pro-B-type natriuretic peptide; NYHA, New York Heart Association; eGFR, estimated glomerular filtration rate; LDL, Low-density lipoprotein; HDL, High-density lipoprotein; LVEF, Left ventricular ejection fraction; CAD, Coronary artery disease; ECL, Electrochemiluminescence. ^*^This study was originally designed as a trial; however, the analyses included here are *post hoc* analyses of men with available baseline plasma testosterone concentrations. ^**^This study was originally designed as a case-control study, but the analyses presented here focus on 264 patients (cases) who were followed for a median of 5.6 year.

Out of these, nine studies were conducted in European countries (19, 22, 23, 30, 33–37), two in Asian countries (20, 31), and one in the United States (32). Nine studies focused on men (19, 20, 22, 31–35, 37), two on women (23, 30), and one included both men and women in a combined analysis (36). Six studies were conducted among patients with (systolic) heart failure (20, 22, 30, 31, 33, 37), two with acute coronary syndrome (19, 34), one with acute myocardial infarction (35), one with acute stroke (35), one with atherosclerotic CVD (32), and one ischemic heart disease (36). Additionally, most had a mean participant age over 50 years (20, 22, 23, 30, 31, 33–37) with median follow-up of 34 months.

Among the six studies included only in the narrative analysis (23, 30, 31, 35–37), the majority were rated as having some concerns (four studies) and two studies were classified as high risk, primarily due to potential confounding bias based on the ROBINS-E. The other six studies were included in the meta-analysis (19, 20, 22, 32–34). Five out of six were classified as having a high risk of bias, mainly driven by potential confounding and missing data (**Supplementary Table 2**).

### Findings of the narrative analysis

In a cohort of 302 women with acute stroke, estradiol emerged as an independent determinant of 1-month mortality (OR: 2.28; 95% CI: 1.27–4.07) (23). In 94 women with heart failure, testosterone deficiency (defined as serum total testosterone <25 ng/dl) was strongly associated with all-cause mortality (HR: 8.33; 95% CI: 5.36–15.11) (30).

The OR (95% CI) for the fourth quartile of free testosterone at admission compared with the lowest quartile was 0.56 (0.40–0.83) (35). Similarly, in a cohort of 618 men with heart failure, higher levels of total testosterone were associated with a lower risk of all-cause mortality (HR: 0.92; 95% CI: 0.86–0.99) (31). Additionally, in 104 men with heart failure, SHBG was associated with a higher risk of CVD mortality (HR: 1.05; 95% CI: 1.02–1.08) (37). In 501 men with systolic chronic heart failure, higher estradiol concentrations were associated with increased mortality risk (HR: 2.40; 95% CI: 1.35–4.28), whereas higher DHEAs levels were associated with a lower risk of death (HR: 0.63; 95% CI: 0.47–0.84) (22). By contrast, other studies did not observe statistically significant associations. For instance, in 191 men with heart failure, higher levels of free testosterone (HR: 0.92; 95% CI: 0.84–1.07), DHEAs (HR: 0.97; 95% CI: 0.91–1.03), and SHBG (HR: 1.06; 95% CI: 0.92–1.23) were not significantly related to all-cause mortality after (33). Similarly, in 175 men with systolic heart failure, neither SHBG (HR: 1.08; 95% CI: 0.88–1.26) nor free testosterone (HR: 0.92; 95% CI: 0.82–1.06) were associated with risk of all-cause mortality (20). Finally, among 434 men and women with ischemic heart disease, a lower total testosterone to estradiol ratio was associated with a higher risk of all-cause mortality (HR: 3.49; 95% CI: 1.24–9.80). These results are reported for the combined population of men and women (36).

### Findings of the meta-analysis

**Figure 2:**
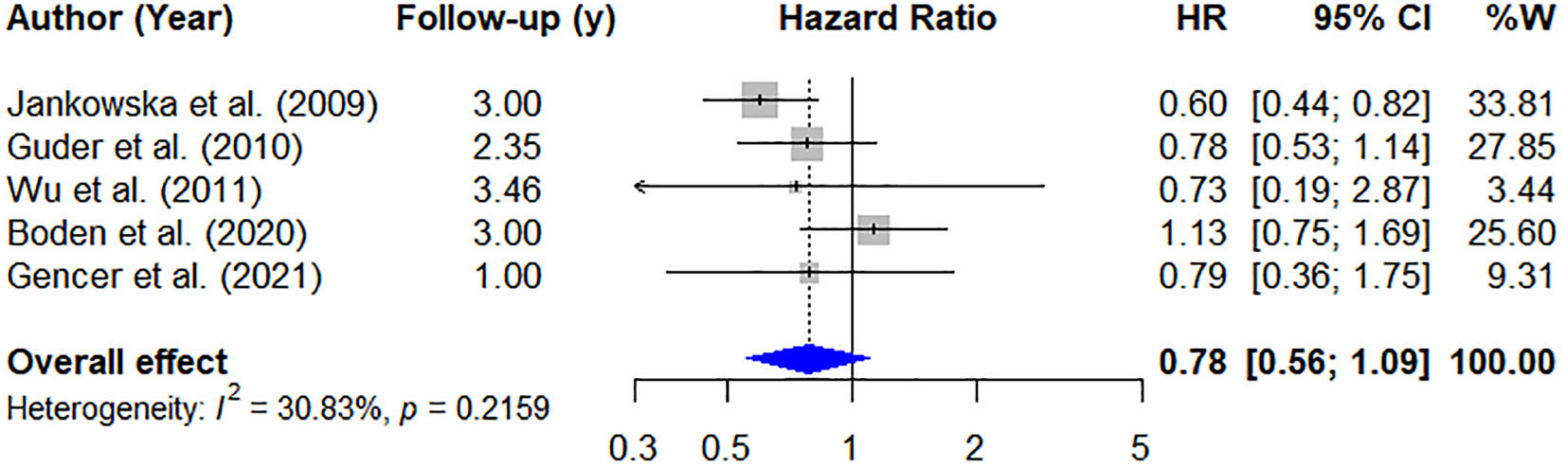
Pooled hazard ratio for total testosterone and all-cause mortality in men with established CVD. y, Year.

Five studies, including a total of 4,039 male participants and 429 all-cause mortality events, were included in the meta-analysis of total testosterone levels with the risk of all-cause mortality in men. The comparison between the highest versus lowest levels of total testosterone showed no significant association with risk of all-cause mortality (HR: 0.78; 95% CI: 0.56–1.09; I² = 30.83%, **Figure 2**). Sensitivity analysis using the leave-one-out method revealed that excluding the study by Boden et al. resulted in a statistically significant association, with no between study heterogeneity (HR: 0.68; 95% CI: 0.53–0.86; I² = 0.0%, **Supplementary Figure 1**).

**Figure 3:**
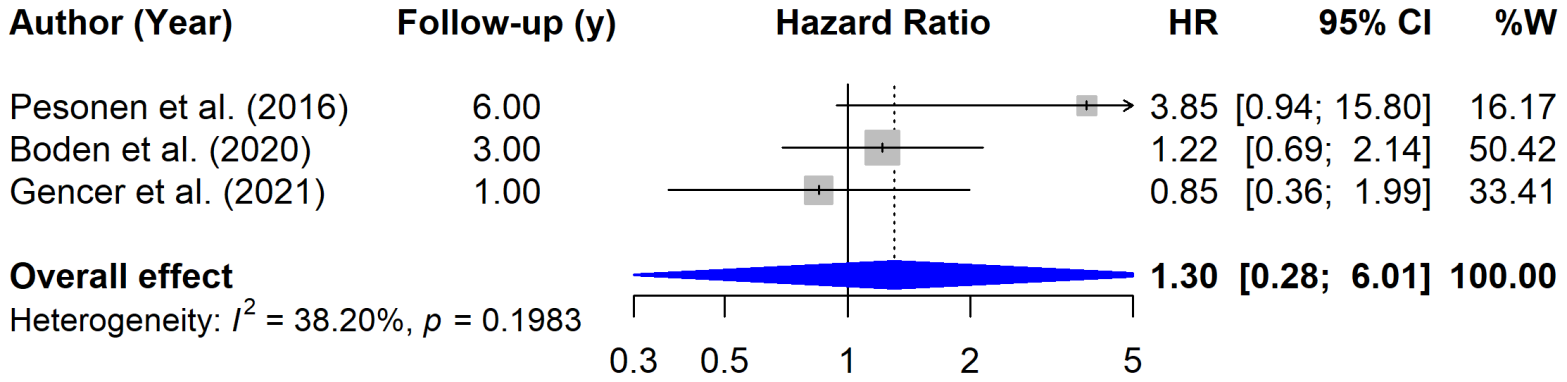
Pooled hazard ratio for total testosterone and CVD mortality in men with established CVD. y, Year.

Three studies, including a total of 3,436 male participants and 135 cardiovascular-related deaths, were included in the meta-analysis examining the association between total testosterone levels and the risk of CVD mortality in men. The analysis found no significant association between total testosterone levels and the risk of CVD mortality (HR: 1.30; 95% CI: 0.28–6.01; I² = 38.20%, **Figure 3**). These findings were not sensitive to the exclusion of any individual study (**Supplementary Figure 2**).

## Discussion

To the best of our knowledge, this is the first systematic review and meta-analysis to examine the association between endogenous sex hormones and SHBG, and the risk of all-cause and CVD mortality in patients with established CVD. No significant association was observed between higher endogenous total testosterone levels and the risk of either all-cause or CVD mortality in men. These findings should be interpreted with caution, as the male patient participants included in the studies varied widely in their underlying conditions and characteristics. Evidence regarding other endogenous sex hormones, SHBG, and studies involving women remains limited and inconclusive.

In the general population, an individual participant data (IPD) meta-analysis of nine prospective cohort studies with at least five years of follow-up found a non-linear association between total testosterone levels and all-cause or CVD mortality. Specifically, all-cause mortality risk increased in men with total testosterone levels below 7.4 nmol/L, while CVD mortality risk rose at levels below 5.3 nmol/L (13). Findings from another systematic review and meta-analysis of prospective cohort studies in the general male population revealed a U-shaped association between endogenous total testosterone levels and the risk of all-cause mortality, whereas no significant association was observed with CVD mortality (14). In our study we were limited by the number of included articles to perform non-linear analysis, which could have provided more insights into the relationship between testosterone and all-cause mortality. Among patients with CVD, a randomized, double-blind, placebo-controlled trial involving 5,246 men with preexisting CVD or at high risk for CVD demonstrated that testosterone replacement therapy (TRT) was noninferior to placebo in relation to the incidence of major adverse cardiac events. Additionally, the occurrence of secondary endpoint events, including any component of the composite of cardiovascular death, nonfatal myocardial infarction, nonfatal stroke, or coronary revascularization was similar between the two groups (38). The recent meta-analysis of 30 randomized controlled trials in hypogonadal men found that TRT did not elevate the risk of cardiovascular events or mortality. These trials included participants with common cardiovascular risk factors, such as diabetes and hypertension (39). Furthermore, an IPD meta-analysis including 3,431 men with hypogonadism found no evidence that TRT increased short- to medium-term cardiovascular risk. Notably, the included randomized trials were not limited to men free of cardiovascular disease at baseline and encompassed participants with pre-existing conditions such as myocardial infarction, coronary heart disease, atherosclerosis, heart failure, and diabetes, among other cardiovascular comorbidities (40).

Several explanations may account for the discrepancies between findings in the general population and patients with established CVD. Studies in the general population typically include healthier individuals with greater hormonal variability, whereas individuals with CVD often have advanced disease states, where hormonal effects may be attenuated by competing risks. Additionally, severe or acute illness can disrupt the hormonal system which may lead to temporary hypogonadism in men (41–44). Further, differences in CVD subtypes across studies may influence hormone-mortality relationship and limited number of studies and/or sample sizes in CVD-specific studies reduce the power to detect associations. While most studies adjust for metabolic factors (for example blood pressure, diabetes, body mass index), residual confounding might be of concern for studies in CVD patients as they often neglect lifestyle confounders such as physical activity and dietary intakes. Also, existing studies in patients with CVD have been constrained by shorter follow-up durations compared to those in the general population, with none providing longitudinal data beyond 10 years.

Several limitations should be acknowledged. This analysis synthesized findings from multiple cohort studies that varied in hormonal assessment methods, sample sizes, follow-up durations, and statistical adjustments. In particular, the cardiovascular status of participants differed across the studies included in the meta-analysis and comprised conditions such as atherosclerotic cardiovascular disease, acute coronary syndrome, and heart failure (including systolic heart failure), which reflect distinct clinical conditions and involve partly different underlying pathophysiological mechanisms. Additionally, the inconsistency in hormone measurement techniques, especially for total testosterone in women and estradiol in postmenopausal women and men, is a notable concern. Methods such as immunoassays are known to be less reliable at low hormone concentrations due to method-specific biases and limited sensitivity. Additionally, we were unable to conduct meta-analyses for women or for endogenous sex steroid hormones other than total testosterone due to the limited number of eligible studies. Additionally, due to the limited number of studies, we were unable to perform any subgroup analyses such as by risk of bias assessment, cardiovascular type, to explore potential sources of heterogeneity or differences between studies. This limitation should be considered when interpreting the pooled results.

Our paper underscores a critical gap in the literature concerning women with CVD. A meta-analysis of observational cohort studies in the general population identified a J-shaped association between total testosterone levels and all-cause mortality in women, and found that higher SHBG levels were linked to increased mortality risk (14). However, the relevance of these findings to women with established CVD (23, 30) remains unclear. In particular, there is a complete lack of data on the association between SHBG and mortality in this population, despite SHBG’s potential role in the risk of chronic diseases independent of sex hormone levels (9–12). Furthermore, considering known sex hormone variations across ethnic groups, there is a need for studies in more diverse populations to better understand ethnic and sex-based differences in hormonal influences on CVD outcomes. Moreover, further research should prioritize the development of clinically relevant reference ranges for hormone levels in patients with CVD and investigate whether hormone-guided therapy could benefit specific high-risk subgroups.

The findings of this review suggest that in individuals with established CVD, the effects of endogenous sex hormones on mortality risk may be less pronounced than in the general population, potentially overshadowed by the underlying disease process. Consequently, the associations observed between sex hormones and mortality risk in general population may not directly translate to patients with established cardiovascular diseases. Clinicians should be cautious when interpreting these findings for secondary prevention, as the evidence in this context remains limited and is largely rated as having a high risk of bias according to ROBINS-E, primarily due to confounding. Furthermore, the notable lack of data on women with CVD presents a significant gap in our understanding of clinical practice. Given the current evidence, there is insufficient justification to incorporate sex hormone levels into routine risk assessment or management strategies for women with CVD. This is particularly relevant considering the cardioprotective effects of estrogen in women (45, 46). More research is needed to determine whether sex hormone levels might offer valuable insights for clinical care in these populations.

## Conclusion

This systematic review and meta-analysis provides a comprehensive overview of the current evidence on this topic; however, it found no evidence of an association between testosterone and risk of all-cause mortality among men. In addition, evidence on other sex hormones and on women remains limited, underscoring an important gap in the existing literature.
