# Supplementary material for: Endogenous sex steroid hormones, sex hormone binding globulin and risk of all-cause and cardiovascular mortality in patients with established cardiovascular diseases: a systematic review and meta-analysis of prospective studies
Source: Front Endocrinol (Lausanne). 2026 Jul 27;17:1878347. doi: 10.3389/fendo.2026.1878347 (PMC13454103; doi:10.3389/fendo.2026.1878347)
Supplement: Supplementary file 1 [file SupplementaryFile1.docx]

**Table S1.** Search strategy

| **Database** | **Search strategy** |
| --- | --- |
| **Embase.com** | ('sex hormone binding globulin'/de OR 'androgen'/de OR 'androgen blood level'/exp OR 'estradiol'/de OR 'estradiol blood level'/de OR 'testosterone'/de OR 'testosterone blood level'/de OR 'dehydroepiandrosterone'/de OR 'dehydroepiandrosterone sulfate'/de OR (androgen* OR 'sex hormone*' OR 'sex steroid*' OR testosterone* OR 'sexual hormone*' OR shbg OR dehydroepiandrosterone* OR androstenolone* OR dhea OR dheas OR 'dehydroepiandrosterone sulfate' OR 'dhea s' OR estradiol* OR oestradiol* OR prasterone*):ab,ti) AND ('mortality'/exp OR 'death'/exp OR 'mortality risk'/de OR 'mortality risk score'/de OR 'cardiovascular disease'/de OR 'coronary artery disease'/de OR 'heart muscle ischemia'/de OR 'heart infarction'/de OR 'Coronary Disease'/de OR 'cerebrovascular accident'/de OR 'heart failure'/de OR (mortalit* OR death* OR fatal* OR 'cardiovascular disease*' OR 'coronary disease*' OR 'coronary artery disease*' OR 'heart disease*' OR 'coronary heart disease*' OR 'ischemic heart disease*' OR 'ischaemic heart disease*' OR 'myocardial ischemia*' OR 'myocardial ischaemia*' OR 'myocardial infarct*' OR 'heart infarct*' OR stroke* OR 'sudden cardiac death' OR 'heart failure*' OR 'cardiovascular event*' OR 'cardiac event*' OR MACE OR MACEs OR survival* OR CVD OR CHD):ab,ti) AND ('cohort analysis'/de OR 'prospective study'/de OR 'follow up'/de OR 'longitudinal study'/de OR 'hazard ratio'/de OR (((hazard* OR odds OR risk* OR inciden*) NEXT/1 (ratio* OR proportion*)) OR cohort* OR prospectiv* OR longitudinal OR follow-up* OR followup* OR followed-up OR population-based OR case-cohort* OR nested case-control* OR 'relative risk*' OR inciden*):ab,ti) NOT ([animals]/lim NOT [humans]/lim) NOT (animal*:ti OR mice:ti OR rat:ti OR rats:ti OR in-vivo:ti OR in-vitro:ti OR tissue:ti OR tissues:ti OR cell*:ti) NOT (([newborn]/lim OR [infant]/lim OR [child]/lim OR [adolescent]/lim) NOT [adult]/lim) NOT ('pregnancy'/exp OR pregnan*:ti OR fetus:ti OR fetal:ti OR foetus:ti OR foetal:ti OR neonatal*:ti OR perinatal*:ti OR maternal*:ti OR infant*:ti OR child*:ti OR newborn*:ti OR pediatric*:ti OR paediatric*:ti OR juvenile*:ti) NOT ([Conference Abstract]/lim OR [Letter]/lim OR [Note]/lim OR [Editorial]/lim OR [Review]/lim) NOT ('systematic review'/exp OR 'meta analysis'/exp OR 'case report'/de OR 'case study'/de OR 'case report*':ti OR 'case stud*':ti OR 'case series':ti OR review*:ti OR 'meta-analysis':ti) |
| **Medline (Ovid)** | (Sex Hormone-Binding Globulin/ OR Androgens/ OR Estradiol/ OR Testosterone/ OR Dehydroepiandrosterone/ OR Dehydroepiandrosterone Sulfate/ OR (androgen* OR sex hormone* OR sex steroid* OR testosterone* OR sexual hormone* OR shbg* OR dehydroepiandrosterone* OR androstenolone* OR dhea OR dheas OR dehydroepiandrosterone sulfate OR dhea-s OR estradiol* OR oestradiol* OR prasterone*).ab,ti.) AND (exp Mortality/ OR exp Death/ OR Cardiovascular Diseases/ OR Coronary Disease/ OR Coronary Artery Disease/ OR Myocardial Ischemia/ OR Myocardial Infarction/ OR exp Stroke/ OR Heart Failure/ OR (mortalit* OR death* OR fatal* OR cardiovascular disease* OR coronary disease* OR coronary artery disease* OR heart disease* OR coronary heart disease* OR ischemic heart disease* OR ischaemic heart disease* OR myocardial ischemia* OR myocardial ischaemia* OR myocardial infarct* OR heart infarct* OR stroke* OR sudden cardiac death* OR heart failure* OR cardiovascular event* OR cardiac event* OR MACE OR MACEs OR survival* OR CVD OR CVDs OR CHD).ab,ti.) AND (Cohort Studies/ OR Prospective Studies/ OR Follow-Up Studies/ OR Longitudinal Studies/ OR (((hazard* OR odds OR risk* OR inciden*) adj1 (ratio* OR proportion*)) OR cohort* OR prospectiv* OR longitudinal OR follow-up* OR followup* OR followed-up OR population-based OR case-cohort* OR nested case-control* OR relative risk* OR (inciden* AND risk*)).ab,ti.) NOT (exp animals/ NOT humans/) NOT (animal*.ti. OR mice.ti. OR rat.ti. OR rats.ti. OR in-vivo.ti. OR in-vitro.ti. OR tissue.ti. OR tissues.ti. OR cell*.ti.) NOT ((exp infant/ OR exp child/ OR adolescent/) NOT (exp adult/)) NOT (exp Pregnancy/ OR pregnan*.ti. OR fetus.ti. OR fetal.ti. OR foetus.ti. OR foetal.ti. OR neonatal*.ti. OR perinatal*.ti. OR maternal*.ti. OR infant*.ti. OR child*.ti. OR newborn*.ti. OR pediatric*.ti. OR paediatric*.ti. OR juvenile*.ti.) NOT (letter* OR news OR comment* OR editorial* OR congres*).pt. NOT ("review"/ OR systematic review/ OR meta-analysis/ OR case reports/ OR case report*.ti. OR case series.ti. OR case stud*.ti. OR review*.ti. OR meta-analysis.ti.) NOT (Hormone Replacement Therapy/ OR replacement.ti OR (hormone adj2 treatment*).ti OR (hormone adj2 therap*).ti) |
| **Cochrane Central Register of Controlled Trials (CENTRAL)** | ((androgen* OR sex NEXT hormone* OR sex NEXT steroid* OR testosterone* OR sexual NEXT hormone* OR shbg OR dehydroepiandrosterone* OR androstenolone* OR dhea OR dheas OR "dehydroepiandrosterone sulfate" OR "dhea-s" OR estradiol* OR oestradiol* OR prasterone*):ab,ti) AND ((mortalit* OR death* OR fatal* OR cardiovascular NEXT disease* OR coronary NEXT/2 disease* OR heart NEXT disease* OR ischemic NEXT heart NEXT disease* OR ischaemic NEXT heart NEXT disease* OR myocardial NEXT ischemia* OR myocardial NEXT ischaemia* OR myocardial NEXT infarct* OR heart NEXT infarct* OR stroke* OR "sudden cardiac death" OR heart NEXT failure* OR cardiovascular NEXT event* OR cardiac NEXT event* OR MACE OR MACEs OR survival* OR CVD OR CHD):ab,ti) AND ((((hazard* OR odds OR risk* OR inciden*) NEXT/1 (ratio* OR proportion*)) OR cohort* OR prospectiv* OR longitudinal OR follow NEXT up* OR followup* OR followed NEXT up OR population NEXT based OR case NEXT cohort* OR nested NEXT case NEXT control* OR relative NEXT risk* OR inciden*):ab,ti) NOT (replacement:ti OR hormone NEXT/2 treatment*:ti OR hormone NEXT/2 therap*:ti) NOT (pregnan*:ti OR fetus:ti OR fetal:ti OR foetus:ti OR foetal:ti OR neonatal*:ti OR perinatal*:ti OR maternal*:ti OR infant*:ti OR child*:ti OR newborn*:ti OR pediatric*:ti OR paediatric*:ti OR juvenile*:ti) |
| **Web of Science Core Collection** | Focus on precision: search terms for sex hormones and CV-mortality, all-cause mortality only in *Title* and *Keyword Plus*  TI=((androgen* OR "sex hormone*" OR "sex steroid*" OR testosterone* OR "sexual hormone*" OR shbg OR dehydroepiandrosterone* OR androstenolone* OR dhea OR dheas OR "dehydroepiandrosterone sulfate" OR "dhea-s" OR estradiol* OR oestradiol* OR prasterone*) AND (mortalit* OR death* OR fatal* OR "cardiovascular disease*" OR coronary NEAR/2 disease* OR "heart disease*" OR "ischemic heart disease*" OR "ischaemic heart disease*" OR "myocardial ischemia*" OR "myocardial ischaemia*" OR "myocardial infarct*" OR "heart infarct*" OR stroke* OR "sudden cardiac death*" OR "heart failure*" OR "cardiovascular event*" OR "cardiac event*" OR MACE OR MACEs OR survival* OR CVD OR CHD)) OR KP=((androgen* OR "sex hormone*" OR "sex steroid*" OR testosterone* OR "sexual hormone*" OR shbg OR dehydroepiandrosterone* OR androstenolone* OR dhea OR dheas OR "dehydroepiandrosterone sulfate" OR "dhea-s" OR estradiol* OR oestradiol* OR prasterone*) AND (mortalit* OR death* OR fatal* OR "cardiovascular disease*" OR coronary NEAR/2 disease* OR "heart disease*" OR "ischemic heart disease*" OR "ischaemic heart disease*" OR "myocardial ischemia*" OR "myocardial ischaemia*" OR "myocardial infarct*" OR "heart infarct*" OR stroke* OR "sudden cardiac death*" OR "heart failure*" OR "cardiovascular event*" OR "cardiac event*" OR MACE OR MACEs OR survival* OR CVD OR CHD))  AND TS=(((hazard* OR odds OR risk* OR inciden*) NEAR/1 (ratio* OR proportion*)) OR cohort* OR prospectiv* OR longitudinal OR "follow up*" OR followup* OR "followed up" OR "population based" OR "case cohort*" OR "nested case control*" OR "relative risk*" OR inciden*) NOT TS=((animal* OR plant* OR rat OR rats OR mouse OR mice OR murine OR pigs OR primate* OR nonhuman*) NOT (human* OR patient*)) Exclude Document Types: Review Articles |
| **PubMed** (publisher) | (Sex Hormone-Binding Globulin[mh] OR Androgens[mh] OR Estradiol[mh] OR Testosterone[mh] OR Dehydroepiandrosterone[mh] OR Dehydroepiandrosterone Sulfate[mh] OR (androgen*[tiab] OR sex hormone*[tiab] OR sex steroid*[tiab] OR testosterone*[tiab] OR sexual hormone*[tiab] OR shbg*[tiab] OR dehydroepiandrosterone*[tiab] OR androstenolone*[tiab] OR dhea[tiab] OR dheas[tiab] OR dehydroepiandrosterone sulfate[tiab] OR dhea-s[tiab] OR estradiol*[tiab] OR oestradiol*[tiab] OR prasterone*[tiab])) AND (Mortality[mh] OR Death[mh] OR Cardiovascular Diseases[mh] OR Coronary Disease[mh] OR Coronary Artery Disease[mh] OR Myocardial Ischemia[mh] OR Myocardial Infarction[mh] OR Stroke[mh] OR Heart Failure[mh] OR (mortalit*[tiab] OR death*[tiab] OR fatal*[tiab] OR cardiovascular disease*[tiab] OR coronary disease*[tiab] OR coronary artery disease*[tiab] OR heart disease*[tiab] OR coronary heart disease*[tiab] OR ischemic heart disease*[tiab] OR ischaemic heart disease*[tiab] OR myocardial ischemia*[tiab] OR myocardial ischaemia*[tiab] OR myocardial infarct*[tiab] OR heart infarct*[tiab] OR stroke*[tiab] OR sudden cardiac death*[tiab] OR heart failure*[tiab] OR cardiovascular event*[tiab] OR cardiac event*[tiab] OR MACE[tiab] OR MACEs[tiab] OR survival*[tiab] OR CVD[tiab] OR CVDs[tiab] OR CHD[tiab])) AND (Cohort Studies[mh] OR Prospective Studies[mh] OR Follow-Up Studies[mh] OR Longitudinal Studies[mh] OR (((hazard*[tiab] OR odds[tiab] OR risk*[tiab] OR inciden*[tiab]) AND (ratio*[tiab] OR proportion*[tiab])) OR cohort*[tiab] OR prospectiv*[tiab] OR longitudinal[tiab] OR follow-up*[tiab] OR followup*[tiab] OR followed-up[tiab] OR population-based[tiab] OR case-cohort*[tiab] OR nested case-control*[tiab] OR relative risk*[tiab] OR (inciden*[tiab] AND risk*[tiab]))) NOT (animals[mh] NOT humans[mh]) NOT (letter[pt] OR news[pt] OR comment[pt] OR editorial[pt] OR congress[pt]) AND publisher[sb] |
| **Google Scholar** | "sex\|sexual hormone\|hormones\|steroids"\|androgen\|testosterone\|estradiol\|oestradiol\|dehydroepiandrosterone\|dhea\|dheas mortality\|death\|fatal\|cardiovascular\|cardiac\|coronary\|ischemic\|heart\|myocardial\|stroke\|survival cohort\|prospective\|"follow-up"\|followup\|longitudinal |

**Table S2.** ROBINS-E judgement for each domain and overall.

| **First Author, year (ref)** | **Bias due to confounding** | **Bias due to exposure assessment** | **Bias due to selection of participants** | **Bias due to post-exposure interventions** | **Bias due to missing data** | **Bias due to measurement of the outcome** | **Bias due to selective reporting of the results** | **Overall judgement** |
| --- | --- | --- | --- | --- | --- | --- | --- | --- |
| Militaru et al., 2010 [1] | Some concerns | Low risk | Low risk | Low risk | Low risk | Low risk | Low risk | Some concerns |
| Pappa et al., 2012 [2] | Some concerns | Low risk | Low risk | Low risk | Low risk | Low risk | Low risk | Some concerns |
| Pascual-Figal et al., 2009 [3] | Some concerns | Low risk | Low risk | Low risk | Low risk | Low risk | Low risk | Some concerns |
| Marra et al., 2023 [4] | High risk | Some concerns | Low risk | Low risk | Low risk | Low risk | Low risk | High risk |
| Raparelli et al., 2022 [5] | Some concerns | Low risk | Low risk | Low risk | Some concerns | Low risk | Low risk | Some concerns |
| Yoshihisa et al., 2018 [6] | High risk | Low risk | Low risk | Low risk | Some concerns | Low risk | Low risk | High risk |
| Boden et al., 2020 [7] | Some concerns | Low risk | Low risk | Low risk | Some concerns | Low risk | Low risk | Some concerns |
| Gencer et al., 2021 [8] | High risk | Low risk | Low risk | Low risk | Some concerns | Low risk | Low risk | High risk |
| Guder et al., 2010 [9] | High risk | Low risk | Low risk | Low risk | Low risk | Low risk | Low risk | High risk |
| Jankowska et al., 2009 [10] | Some concerns | Low risk | Low risk | Low risk | High risk | Low risk | Low risk | High risk |
| Pesonen et al., 2016 [11] | High risk | Low risk | Some concerns | Low risk | Some concerns | Low risk | Low risk | High risk |
| Wu et al., 2011 [12] | High risk | Low risk | Some concerns | Low risk | Some concerns | Low risk | Some concerns | High risk |

**Figure S1.** Leave-one-out analysis evaluating the association between total testosterone levels and all-cause mortality in men with established CVD.

**Figure S2.** Leave-one-out analysis evaluating the association between total testosterone levels and CVD mortality in men with established CVD.

References

1.	Militaru, C., et al., *Serum testosterone and short-term mortality in men with acute myocardial infarction.* Cardiology Journal, 2010. **17**(3): p. 249-53.

2.	Pappa, T., et al., *Estradiol levels predict short-term adverse health outcomes in postmenopausal acute stroke women.* European Journal of Neurology, 2012. **19**(10): p. 1300-4.

3.	Pascual-Figal, D.A., et al., *Sex hormone-binding globulin: a new marker of disease severity and prognosis in men with chronic heart failure.* Revista Espanola de Cardiologia, 2009. **62**(12): p. 1381-7.

4.	Marra, A.M., et al., *Testosterone deficiency independently predicts mortality in women with HFrEF: insights from the T.O.S.CA. registry.* ESC heart failure, 2023. **10**(1): p. 159-166.

5.	Raparelli, V., et al., *Testosterone-to-estradiol ratio and platelet thromboxane release in ischemic heart disease: the EVA project.* Journal of Endocrinological Investigation, 2022. **09**: p. 09.

6.	Yoshihisa, A., et al., *Relation of Testosterone Levels to Mortality in Men With Heart Failure.* American Journal of Cardiology, 2018. **121**(11): p. 1321-1327.

7.	Boden, W.E., et al., *Testosterone concentrations and risk of cardiovascular events in androgen-deficient men with atherosclerotic cardiovascular disease.* American Heart Journal, 2020. **224**: p. 65-76.

8.	Gencer, B., et al., *Prognostic value of total testosterone levels in patients with acute coronary syndromes.* European Journal of Preventive Cardiology, 2021. **28**(2): p. 235-242.

9.	Guder, G., et al., *Low circulating androgens and mortality risk in heart failure.* Heart, 2010. **96**(7): p. 504-9.

10.	Jankowska, E.A., et al., *Circulating estradiol and mortality in men with systolic chronic heart failure.* JAMA, 2009. **301**(18): p. 1892-901.

11.	Pesonen, E., P. Pussinen, and I. Huhtaniemi, *Adaptation to acute coronary syndrome-induced stress with lowering of testosterone: a possible survival factor.* European Journal of Endocrinology, 2016. **174**(4): p. 481-9.

12.	Wu, H.Y., et al., *Testosterone level and mortality in elderly men with systolic chronic heart failure.* Asian Journal of Andrology, 2011. **13**(5): p. 759-63.
